# On psychiatry and psychology

**DOI:** 10.1192/j.eurpsy.2021.1302

**Published:** 2021-08-13

**Authors:** E. Neu, M. Michailov, U. Welscher, M. Schratz, G. Weber

**Affiliations:** 1 Pharmaco-physiology, Inst. Umweltmedizin (IUM) c/o ICSD/IAS e.V., POB 340316, 80100 M. (Int.Council Sci.Develop./Int.Acad.Sci. Berlin-Innsbruck-Muenchen-NewDelhi-Paris-Sofia-Vienna), Muenchen, Germany; 2 School Of Education (dean), Univ. Innsbruck, Innsbruck, Austria; 3 Fac. Psychology, Univ. Luxemburg & Vienna, Vienna, Austria

**Keywords:** cyclophrenia, integrative psychiatry, psychophysiology, Epilepsy

## Abstract

**Introduction:**

Psychiatry is fundamental interdisciplinary medical science with essential importance for enormous health-problems of humanity. Creation of integrative-psychiary in context of multidimensional&holistic medicine, founded by HIPPOCRATES-GALENUS-HUA T’UA-AVICENA-PARACELSUS is necessary to counteract disastrous human health-situation. Psychiatry needs new integrative therapy-models considering application of psychopharmacotherapy as well as practices of psycho-somatic (Th.v.UEXKÜLL) and somato-psychic theories (Y.IKEMI). Emperor AKIHITO during Opening-Ceremony of ICPM-2005-Kobe appointed to consider “total symptoms of mind-body, seeking ways of holistic care”.

**Objectives:**

REFERENCES. PSYCHIATRY: EPA-2020-virtual/Madrid, Eur.Psychiatry 63S, EPP0834/5+EPV0581/1470; EPA-2019-Warsaw, Eur. Psychiatry 56S,S689; EPA-2018-Nice, Eur.Psychiatry 48/S1, S623&567&662. WPA-2019-Lisbon, E-Poster WCP19-2137, -1822, -1839; 2018-Mexico-City, Abs.-Book WCP18-0584/-0625/-0643/-0654. 2011-Buenos-Aires,AB:PO1.200. PSYCHOLOGY: EFPA-2019-Moscow, Abs.-Book 1529,1530,1549. IUPsyS-2012-Cape-Town, IntJPsychol 47:407; -2008-Berlin, 43/3-4:154, 248,615,799; -2004-Beijing, AB:49,587. PSYCHOSOMATICS: ICPM-2017-Beijing, AB:ID: 648493,648895,648749,648878; -2005-Kobe, J.Psychosom.Res. 58:85-86.

**Methods:**

Evaluation of psychic-“polar-attitude-list”/physiological-parameters: heart-rate, blood-pressure,etc. from patients/probands after training by occidental/oriental practices (Music-/Yogatherapy/others) (ref.).

**Results:**

Observations demonstrate strong positive influence after music[1], respiratory[2], hatha-yoga[3] therapies. Items of psycho-physiological (relaxed), emotional (tranquil/happy), cognitive (few/ordered thoughts), voluntary (active/spontaneous), social (open/assertive), consciousness (clear/sleepy) categories are significantly positive changed 25-50%. The 3-therapies have specific psychic-effects,e.g. items “relaxed/tranquil” after respiratory- (+45/50%) and music- (+20/5%), also item “open” after music-therapy (+25%) are positive, but negative after respiratory-therapy (-20%). Psychic-effects are correlated with positive physiological-ones,e.g. heart/respiratory-frequency decreased 25-30%, voluntary-apnoea prolonged 55%. Mountain-altitude (>2000-3000m), hypothermia (<20 to 0°C) influenced positively psychic/physiological-parameters,e.g. heart-rate/blood-pressure decrease (n=125,P<0.05-0.01).

**Conclusions:**

Different methods of integrative psychiatric therapy are with preference,e.g. for depression is suitable respiratory/physical-training, also hypothermia&high-mountain therapy (activation-euphoria), for mania:music-therapy (inhibitory-effect). Systematically research about single/combined therapies is necessary,e.g. for epilepsy: Respiratory-therapy/hypothermia,etc. could help patients (hypo-/hypercapnia: inhibitory/excitatory effects on CNS-structures).

